# Does the first trimester of pregnancy induce alterations in the walking pattern?

**DOI:** 10.1371/journal.pone.0209766

**Published:** 2019-01-16

**Authors:** Wanda Forczek, Agata Masłoń, Barbara Frączek, Marta Curyło, Marcin Salamaga, Agnieszka Suder

**Affiliations:** 1 Section of Biomechanics, Faculty of Physical Education and Sport, University of Physical Education, Krakow, Poland; 2 Section of Rehabilitation in Orthopaedics, Department of Clinical Rehabilitation and Laboratory of Pathology of the Musculoskeletal System, Faculty of Motor Rehabilitation, University of Physical Education, Krakow, Poland; 3 Section of Human Nutrition, Faculty of Physical Education and Sport, University of Physical Education, Krakow, Poland; 4 Section of Rehabilitation in Internal Diseases, Department of Clinical Rehabilitation, Faculty of Motor Rehabilitation, University of Physical Education, Krakow, Poland; 5 Department of Statistics, Cracow University of Economics, Krakow, Poland; 6 Section of Anatomy, Department of Physiotherapy, Faculty of Motor Rehabilitation, University of Physical Education, Krakow, Poland; West Virginia University, UNITED STATES

## Abstract

**Introduction:**

From among many studies observing the walking pattern throughout pregnancy, only two items monitor the influence of pregnancy on the movement system during gait considering the period before gestation.

**Research question:**

Does the women’s gait pattern at the end of the first trimester undergo changes in comparison to body movement pattern before pregnancy?

**Methods:**

All subjects who met the inclusion criteria gave signed and informed consent before the study. Two experimental sessions were arranged according to the same protocol: (P0) before pregnancy and (P1) at the end of the first trimester of pregnancy (12^th^ week of gestation). At first the anthropometric measures were taken. Then, walking trials at a self-selected speed along a walkway were registered with Vicon 250 (Oxford Metrics Ltd.; Oxford, UK) and FreeMED force platform (Sensor Medica, Italy).

**Results:**

An analysis of anthropometric parameters in 12^th^ pregnancy week demonstrated significant changes in mean values of waist circumference and waist to hip ratio as well as waist to height ratio indexes compared to the results before pregnancy. No significant differences were found in the basic kinematic gait parameters between experimental conditions. Significant increase of mean inter—ankle distance during double support phase occurred during the first trimester of pregnancy. Also, the ratio of the ankle separation width to the pelvic width was noticeably higher in gestation. Then, angular changes of the pelvis in coronal and transverse planes throughout gait cycle during pregnancy demonstrated significant differences compared to those measured before pregnancy. At the same time in the first trimester of pregnancy no adaptive changes in the pattern of feet loading take place.

**Significance:**

Since our study is of longitudinal character, in the course of pregnancy we expect compensatory mechanisms more clearly demonstrated. Therefore, we hope to identify a strategy of the gravid body progression in space.

## Introduction

The issue concerning locomotion of pregnant women has become an area of interest for many scientists who indicate existence of different adaptation mechanisms of the movement system and a strategy of moving woman’s body in that physiological condition [1, 2, 3]. Adaptation of the mother’s organism to the developing foetus takes place for 40 weeks and concerns both sexual organs and functioning of the hormonal, cardio-vascular, respiratory and musculoskeletal systems. Comparison of the studies showed different periods of pregnancy when the registrations of women's gait were performed (As described in detail previously [4]). However, an analysis of literature showed only two items monitoring the influence of pregnancy on the movement system reactions during gait considering the period before [5, 6]. Some of the researchers found a feigned solution to the problem by monitoring women’s gait from the first semester and treating the obtained results as a reference point for subsequent measurements following the advance of pregnancy [7, 8, 9, 10]. However, it is still unclear whether changes in a woman’s body induced in the first trimester affect their movement system and method of realising their gait. The first trimester that lasts from the first day after the end of the last menstruation till the 12^th^ week of pregnancy is a period of changes in the hormonal system, and the level of some hormones may affect, among others, structure of movements [11]. There is an initial increase of relaxin levels until the peak value in the 12^th^ week followed by a decline until the 17^th^ week. Relaxin is known to remodel pelvic connective tissue and activate the collagenolytic system. This, in turn, leads to laxity in the ligamentous and connective tissue, which is the functional adaptation to the foetus developing in the womb [12]. That is why it is essential to understand any potential effects of pregnancy on gait pattern, especially if increased incidence of falls and musculoskeletal pain occurrence during pregnancy is considered [13, 14].

### Research question

The aim of the work was to perform a kinematic analysis of the gait pattern in the context of changes in the movement system connected with foetus development in the first trimester of pregnancy. The main research question was then as follows: Does the women’s gait pattern in the first trimester of pregnancy undergo changes in comparison to the movement scheme before pregnancy?

## Material and methods

### Subjects

The study was carried out in the Biomechanics Laboratory at the University of Physical Education in Krakow. The recruitment process for the study lasted two years (2015–2017). The participants were recruited via direct contact and flyers placed in hospitals or gynaecological clinics and they volunteered to participate in the study. Inclusion criteria aimed to involve only healthy 20–40 year-old women with initial body mass index (BMI) between 18.5 and 24.99 kg /m^2^ who were planning a child in the near future, but not earlier than one year since the last pregnancy. The exclusion criteria included significant medical history concerning previous orthopaedic or neurological injuries. All subjects who met the abovementioned criteria gave signed and informed consent before the beginning of the study. The study was approved by the Regional Bioethics Committee in Krakow (registration no. 139/KBL/OIL/2011). The research were conducted according to the scientific studies ethic principles stated in the Helsinki Declaration.

The sample consisted of 35 subjects who participated in the first experimental session. However, only 15 women from the initial sample become pregnant, and thus, when it was confirmed that they were free of any medical contraindications, they were qualified to the second examination: 8 subjects—primigravid, 6 subjects—second pregnancy and one—third pregnancy. All pregnancies were singleton. The average age in the group, at the time when the study started, was 30.2 ± 3.05 years, body height 166.64 ± 4.06 cm, body weight 59.3 ± 7.7 1 kg, and BMI 21.30 ± 2.24 kg/m^2^. Both experimental sessions were arranged according to the same protocol: (P0) before pregnancy and (P1) at the end of the first trimester of pregnancy (12^th^ week of gestation). At first, the anthropometric measures were taken (Table 1). Then, walking trials at a self-selected speed along a walkway were registered with Vicon 250 (Oxford Metrics Ltd.; Oxford, UK) and FreeMED force platform (Sensor Medica, Italy). During the experimental sessions each woman was wearing tight-fitting shorts and a t-shirt.

**Table 1:** Anthropometric characteristics and indexes of the examined women before pregnancy (PO) and in the 1^st^ trimester of gestation (P1).

Variables		Mean ± SD	t	p	Confidence interval	
					Upper Bound	Upper Bound
**BH** [cm]	**P0**	166.6 ± 4.07	0.72	0.48	-0.38	0.77
	**P1**	166.8 ± 4.28				
**BW** [kg]	**P0**	59.30 ± 7.72	1.36	0.19	-0.64	2.88
	**P1**	60.42 ± 6.73				
**MUAC** [cm]	**P0**	25.67 ± 2.24	-0.50	0.62	-0.67	0.41
	**P1**	25.54 ± 2.17				
**WC** [cm]	**P0**	72.27 ± 6.11	3.78	0.00*	2.35	8.52
	**P1**	77.70 ± 4.16				
**HC** [cm]	**P0**	93.57 ± 4.59	1.20	0.25	-0.94	3.34
	**P1**	94.77 ± 4.79				
**IC-IC** [cm]	**P0**	27.17 ± 1.45	0.61	0.55	-0.58	1.05
	**P1**	27.40 ± 1.35				
**IS-IS** [cm]	**P0**	25.25 ± 1.32	0.00	1.00	-0.64	0.64
	**P1**	25.25 ± 1.56				
**EC** [cm]	**P0**	21.11 ± 1.17	2.47	0.03*	0.08	1.21
	**P1**	21.75 ± 1.55				
**BMI** [kg*m^-2^]	**P0**	21.31 ± 2.24	1.09	0.29	-0.36	1.10
	**P1**	21.68 ± 2.01				
**WHR**	**P0**	0.77 ± 0.04	3.21	0.01*	0.02	0.08
	**P1**	0.82 ± 0.06				
**WHtR**	**P0**	0.43 ± 0.03	-3.68	0.00*	-0.05	-0.01
	**P1**	0.47 ± 0.03				
**TER**	**P0**	1.08 ± 0.25	1.00	0.33	-7.62	21.03
	**P1**	7.78 ± 25.8				
**%FAT**	**P0**	26.27 ± 4.61	1.05	0.31	-5.47	15.92
	**P1**	31.49 ± 19.7				

BH—body height, BW—body weight, MUAC—mid-upper arm circumference, WC—waist circumference, HC—hip circumference, IC-IC–intercristal diameter, IS-IS–interspinous diameter, EC–external conjugate diameter, BMI—Body mass index, WHR–waist to hip ratio, WHtR–waist to height ratio, TER—trunk to extremity ratio, %FAT—percentage of body fat.

* Asterisks denote significant differences between sessions (p< 0.05).

### Study protocol

#### Anthropometric measurements

For the purpose of this study, the following were analysed out of 22 somatic features obtained in the anthropometric measurements: BH, body height (Basis–vertex, measured without shoes, in standing position to the nearest 0.1 cm, with the head in the Frankfurt plane, using a stadiometer), BW, body weight (measured to the nearest 0.1 kg, using a clinical balance scale), MUAC, mid-upper arm circumference (measured at the mid-point between the acromion and the olecranon process), WC, waist circumference (measured to the nearest 0.1 cm by using an anthropometric tape in the narrowest place on the waist between the lower edge of costal arch and the upper edge of iliac crest with the subjects in standing position, recorded at the end of a gentle expiration) and HC, hip circumference (at the level of trochanter major of femoral bone). The pelvis dimensions were assessed based on: IC-IC, intercristal diameter (between the highest points of crests of pelvic girdle), IS-IS, interspinous diameter (between spina iliaca anterior superior) and EC, external conjugate diameter (between anterior superior edge of symphysis pubis and the fossa under the spinous process of the fifth lumbar vertebra), measured by a large spreading calliper. Six following skinfold thicknesses were chosen to be tested in this study: triceps (TRC): a vertical skinfold taken halfway on the back of the upper arm, above triceps muscle, biceps (BIC): a vertical skinfold taken halfway on the upper arm, above biceps muscle, subscapular (SSC): a diagonal skinfold taken under low angle of scapula, abdominal (ABD): a transversal skinfold taken about 1 cm down and 5 cm from umbilicus, suprailiac (SIC): a diagonal skinfold taken in the axillary line, above upper edge of iliac crest, medial calf (CLF): a vertical skinfold taken on the back of the lower leg, slightly under popliteal fossa, in standing position, with the leg slightly flexed at knee (foot rested on toes) and muscles relaxed. They got measured with Harpenden skinfold calliper with 10g/mm^2^ pressure force on the left side of the body following the recommendations of the International Biological Program [15]. The data quality was assured by an extensive training, skinfolds were measured by the same person (A.S.), using the same Harpenden skinfold calliper, calibrated before each series of tests in a specialized workshop.

Several indexes were derived from the anthropometric measurements. Body mass index (BMI) was calculated as mass (kg) divided by height squared (m^2^), waist-hip ratio (WHR) was calculated as WC divided by hip circumference. Waist circumference and waist-to-height ratio (WHtR) were applied to assess the type of adipose tissue distribution. WHtR was calculated by dividing waist circumference (in cm) by height (in cm) x 100. The sum of the three trunk skinfold thicknesses (subscapular, abdominal, and suprailiac) (TTS) was used as the indicator of central fatness, and the sum of the three extremity skinfold thicknesses (triceps, biceps, medial calf) (TES) as the indicator of peripheral fatness. Trunk to extremity ratio (TER), the factor being the relation of the sum of the TTS to the sum of the TES thicknesses, was also calculated. Percentage of body fat (%FAT) was estimated with Durnin and Womersley [16] equation, used the log sum of four skinfold thicknesses (TRC, BIC, SSC, SIC).

#### Gait registration

Five video cameras emitting infrared light, reflected by the markers placed on the subject’s skin were used to register locomotion. Reflective markers were placed over the standard anatomical landmarks according to the Golem model, which required 39 markers (4 on the head, 4 on the trunk, 3 on the pelvis and 7 on each of the upper and lower limbs) as previously described in [6]. The system was calibrated by a 500 mm wand. Calibration was accepted if the standard deviation of the wand length measures was below 0.5 mm. After the calibration of the measuring system, the women were asked to walk barefoot at a self-selected speed across the room on the ground covered with a special surface. Overall, during one session the studied woman covered the distance of about 50 meters with short intervals (about 1 minute) between sections. Gait initiation and termination strides were excluded from the analysis. In all patients at least 10 gait cycles for each leg (20 steps) were registered and analysed. Kinematic parameters were collected at 120 Hz using a 3D motion analysis system (Vicon 250; Oxford Metrics Limited, Oxford, United Kingdom). The analysed parameters were averaged over all trials of all walking subjects. Spatio-temporal gait parameters were measured as proposed by Perry [17]: Double support time was measured as the period during both feet contact with the floor in one walking cycle, corresponding to the period between the initial contact of the heel of one foot and the toe off of the contralateral foot. The stride length was the distance between three consecutive heel strikes. The step cadence was the number of steps per minute. Temporal variables were calculated as normalized values according to total cycle time. For statistical purposes, all repetitions of temporal and spatial variables performed during each cycle were included in the sample.

Additionally, the position of pelvis was observed in three planes, and the mean width of the base of support (BOS) was determined during the double support phase in two manners:

- first: calculated as the mean horizontal line which contains projections of the ANK (markers placed on the left and right lateral malleolus, respectively) (Inter-ANK Distance—IAD) [1];
- second: calculated as the mean horizontal line which contains projections of the MT5 (markers placed on the fifth metatarso-phalangeal joint of the right and left feet, respectively) (Inter-MT5 Distance–IMD). We believe that this measurement more accurately reflects functional BOS during gait, considering the positioning of the feet on the ground (authors' suggestion).

The normalized dynamic BOS width was calculated as the mean width between the ankles during double support (N IAD) divided by the pelvic width (IS-IS) [1].

#### Assessment of the feet loading pattern

Assessment of the feet loading pattern during gait was made using the FreeMED force platform (Sensor Medica, Italy). The examination consisted of passing along the 2.4 metres long measuring path (divided into 40x40 centimetres measuring platform and 2-metre-long platform excluded from measurement, of the same structure and height, which created one, continuous surface) with gait speed and rhythm natural for a given individual. The computer recorded the fluctuations of forces acting on individual sensors in platform-foot contact in real time, and the signal was transferred from the sensors to the computer which stored it in a file. The starting point was determined in such a way that, regardless of the step length, the foot could achieve full contact with the platform at least at the third step (‘midgait technique’) [18]. The examination continued until 3 correct footprints for each side were achieved and the most repeated footprints were used for the analysis. In order to assess force distribution and contact area of the specific foot parts, the obtained picture was automatically divided into 9 regions (medial (1) and lateral (2) heel, medial (3) and lateral (4) foot arch, first (5), second-third (6), and fourth-five (7) metatarsal bones heads, hallux (8), and lesser toes (9). Three indicators were assessed, i.e. Indicator 1: DAI–dynamic longitudinal arch index according to Cavanagh [19] (ratio of medial and lateral foot arch contact area (region 3 and 4) to a sum of the whole foot contact area, excluding toes (regions 1 to 7) [–19]; Indicator 2: FRI—forefoot [%] and rearfoot [%] loading ratio; and Indicator 3: MLI—ratio of medial [%] and lateral [%] foot loading. Moreover, two parameters: an average (from all of the three footprints) contact area [cm^2^] and maximal pressure [gr/cm^2^] for each region were assessed.

### Statistical analysis

The following statistical methods were used: selected descriptive statistics, *t* test for dependent groups (paired sample t-test), and 95 percent confidence interval for mean ranges of motion, or mean values over a portion of the gait cycle for the kinematic data were compared between conditions with the use of paired sample t-tests at a 5 percent significance level. Before using parametric tests, normality of distribution for all variables was checked. For this purpose, Shapiro-Wilk test and corrected Kolmogorov-Smirnov test (Lilliefors test) were applied. The test results at the 5 percent significance level confirmed (almost in all cases) the normality of the variables distribution. With the help of paired sample t-test, the significance of the differences between variables values in the first trimester of pregnancy and the pre-pregnancy period was checked.

## Results

### A comparative analysis of anthropometric parameters before pregnancy and in the 1^st^ trimester of gestation

An analysis of anthropometric parameters in the 12^th^ week of pregnancy indicated significant changes in mean values of waist circumference, waist to hip ratio (WHR) and waist to height ratio (WHtR) indexes as well as in mean values of external conjugate in comparison to the results obtained before pregnancy. The remaining anthropometric parameters from the wide research scheme did not change significantly in the analysed period of pregnancy (Table 1).

### A comparative analysis of kinematic gait parameters before pregnancy and in the 1^st^ trimester of gestation

#### Time and distance gait parameters

No significant differences were found in the basic kinematic gait parameters between experimental conditions (Table 2).

**Table 2:** Gait parameters before pregnancy (P0) and in the 1^st^ trimester of gestation (P1).

Variables		Mean ± SD	t	p	Confidence interval	
					Upper Bound	Upper Bound
**Cad** [step/min]	**P0**	118.7 ± 8.4	1.49	0.16	-0.52	2.88
	**P1**	119.9 ± 8.3				
**DS** [s]	**P0**	0.20 ± 0.04	-0.50	0.62	-0.02	0.01
	**P1**	0.20 ± 0.03				
**SS** [s]	**P0**	0.40 ± 0.02	-0.48	0.63	-0.01	0.01
	**P1**	0.40 ± 0.02				
**FO** [%]	**P0**	0.60 ± 0.01	0.04	0.97	-0.01	0.01
	**P1**	0.60 ± 0.01				
**OFC** [%]	**P0**	0.50 ± 0.00	0.58	0.57	-0.00	0.00
	**P1**	0.50 ± 0.00				
**OFO** [%]	**P0**	0.10 ± 0.01	-0.37	0.71	-0.01	0.01
	**P1**	0.10 ± 0.01				
**SL** [m]	**P0**	1.41 ± 0.12	0.16	0.87	-0.03	0.04
	**P1**	1.41 ± 0.11				
**ST** [s]	**P0**	1.02 ± 0.07	-1.61	0.13	-0.02	0.00
	**P1**	1.00 ± 0.07				
**v** [m/s]	**P0**	1.39 ± 0.16	1.03	0.32	-0.02	0.05
	**P1**	1.41 ± 0.16				

Cad-Cadence, DS-Double Support, SS -Single Support, FO—Foot Off, OFC -Opposite Foot Contact, OFO -Opposite Foot Off, SL -Stride Length, ST -Stride Time, v—velocity

Stride Length and Cadence remained similar in both experimental sessions, which means that Walking Velocity of women around the 12^th^ week of gestation was similar to the one before pregnancy (1.40 m/s). No significant changes were observed in terms of foot contact time with the ground.

#### Base of support (BOS)

Significant increase of the mean inter—ankle distance (p = 0.003) during double support phase was found during the first trimester of pregnancy (Table 3). Also the ratio of the ankle separation width to the pelvic width (N IAD) was noticeably higher in gestation.

**Table 3:** Base of support measures before pregnancy (P0) and in the 1^st^ trimester of gestation (P1).

Variables		Mean ± SD	t	p	Confidence interval	
					Upper Bound	Upper Bound
**IAD** [mm]	**P0**	149 ± 13	3.61	0.00*	3.97	15.59
	**P1**	159 ± 14				
**IMD** [mm]	**P0**	176 ± 18	1.54	0.14	-2.15	13.24
	**P1**	182 ± 15				
**N IAD**	**P0**	0.60 ± 0.06	2.87	0.01*	0.01	0.06
	**P1**	0.64 ± 0.06				

IAD—inter-ankle distance; IMD—inter 5th metatarsal distance; N IAD—normalized inter-ankle distance

* Asterisks denote significant differences between sessions (p< 0.05)

**Fig 1:**
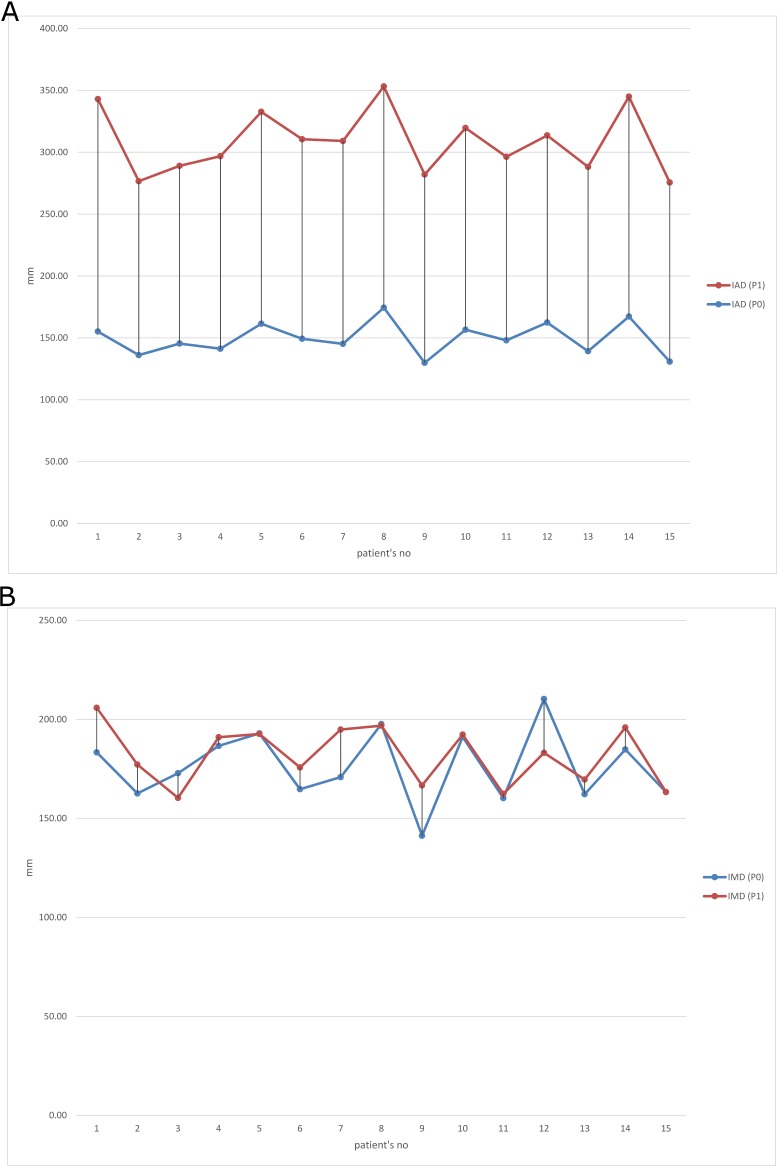
**Individual measures of the Base Of Support (BOS) before pregnancy (P0) and in the 1^st^ trimester of gestation (P1):** a. Inter-ankle distance (IAD) b. Inter 5th metatarsal distance (IMD).

The figures below (Fig 1A and 1B) illustrate individual results of the BOS measures for each woman showing significant changes in IAD and almost no changes in IMD values between P0 and P1 measurements.

#### Pelvic excursions

The authors were interested in the behaviour of the pelvis during walking. Thus, at first the average pelvic range of motion was observed in three planes of movement: before pregnancy (P0) and in the first trimester of gestation (P1). However, no significant changes in pelvic tilt (p = 0.06) and obliquity range of motion (p = 0.10) were found (Table 4).

**Table 4:** Pelvic ranges of motion (ROM) in three planes before pregnancy (P0) and in the 1^st^ trimester of gestation (P1).

Pelvic ROM		Mean ± SD	t	p	Confidence interval	
					Upper Bound	Upper Bound
**Tilt** [deg]	**P0**	2.22 ± 0.60	2.02	0.06	-0.02	0.63
	**P1**	2.52 ± 0.50				
**Obliquity** [deg]	**P0**	14.74 ± 2.97	-1.73	0.10	-2.02	0.21
	**P1**	13.84 ± 2.32				
**Rotation** [deg]	**P0**	22.58 ± 4.53	-1.49	0.16	-2.34	0.42
	**P1**	21.62 ± 4.23				

In case of Pelvic Rotation ROM variable distribution, no conformity to the normal distribution was found (Shapiro-Wilk test results, Kołmogorow test results and Lilliefors test results are statistically significant at the 0.05 significance level) therefore, the nonparametric Wilcoxon test for paired samples was applied in the comparative analysis. Its result was Z = 4.627 (p<0.05), which indicates statistical significance of differences in pelvis motion in transverse plane in the 1^st^ trimester compared with the situation before pregnancy.

**Fig 2:**
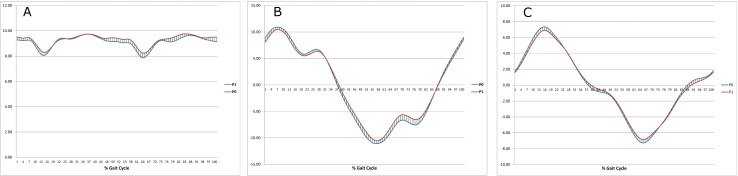
a. Pelvic tilt before pregnancy (P0) and in the 1^st^ trimester of gestation (P1) during gait cycle, b. Pelvic obliquity before pregnancy (P0) and in the 1^st^ trimester of gestation (P1) during gait cycle, c. Pelvic rotations before pregnancy (P0) and in the 1^st^ trimester of gestation (P1) during gait cycle.

Then instantaneous values of the pelvic angle were observed in three planes in two measuring sessions (Fig 2A, 2B and 2C).

The analysis revealed no significant differences in the angular changes of the pelvis in sagittal plane between two experimental conditions (Fig 2A).

However, angular changes of the pelvis in coronal and transverse plane throughout gait cycle during pregnancy demonstrated some significant differences compared to those measured before gestation (Fig 2B and 2C, respectively).

Statistically significant differences were found between angular values of pelvis in coronal plane in stance phase between 10–14% Gait Cycle (GC) and 39–41% GC. The differences turned out to be significantly higher for the tests before pregnancy.

Statistically significant angular values of pelvis in transverse plane were observed in the 1^st^ trimester for the subsequent sections of the gait cycle: the beginning and end of the stance phase (1–3%GC, 47–54%GC, respectively) and a half of the swing phase (68–81%GC).

### A comparative analysis of the foot loading during gait before pregnancy and in the 1^st^ trimester of gestation

The conducted analysis demonstrated that in the 1^st^ trimester there are no adaptive alterations in the feet loading pattern during gait (Table 5).

**Table 5:** Parameters of foot loading during gait before pregnancy (P0) and in the 1^st^ trimester of gestation (P1).

Variables			t	p	Confidence interval	
		Mean ± SD			Upper Bound	Upper Bound
**DAIR**	**P0**	0.15 ± 0.08	0.65	0.52	-0.01	0.03
	**P1**	0.16 ± 0.06				
**DAIL**	**P0**	0.15 ± 0.07	-0.82	0.42	-0.04	0.02
	**P1**	0.14 ± 0.05				
**FRIR**	**P0**	1.62 ± 0.21				
	**P1**	1.53 ± 0.16	-1.82	0.09	-0.20	0.02
**FRIL**	**P0**	1.68 ± 0.21				
	**P1**	1.68 ± 0.17	-0.02	0.99	-0.16	0.16
**MLIR**	**P0**	1.23 ± 0.33				
	**P1**	1.12 ± 0.27	-1.28	0.22	-0.30	0.08
**MLIL**	**P0**	0.98 ± 0.24				
	**P1**	1.03 ± 0.25	0.62	0.54	-0.13	0.23
**CAR** [cm^2^]	**P0**	49.9 ± 6.70				
	**P1**	48.6 ± 4.94	-1.72	0.11	-3.06	0.34
**CAL** [cm^2^]	**P0**	49.9 ± 7.13				
	**P1**	49.31 ± 5.68	-0.71	0.49	-2.69	1.36
**MPR** [gr/cm^2^]	**P0**	1625 ± 201				
	**P1**	1621 ± 176	-0.07	0.94	-98.83	92.26
**MPL** [gr/cm^2^]	**P0**	1591 ± 174				
	**P1**	1620 ± 184	0.64	0.53	-67.48	124.91

DAI–dynamic longitudinal arch index according to Cavanagh (ratio of medial and lateral foot arch contact area (region 3 and 4) to a sum of the whole foot contact area, excluding toes (regions 1 to 7) [19] FRI–index of forefoot [%] and rearfoot [%] loading ratio; MLI—index of medial [%] and lateral [%] foot loading ratio; CA—an average (from all of the three footprints) contact area, MP—maximal pressure; R–right foot; L–left foot.

Both feet arches and their contact area with the ground as well as loads distribution they experienced did not change statistically significantly in the analysed period.

## Discussion

Usually researchers are focused on the investigation of the impact of pregnancy on women's manner of walking assuming that the gait pattern in the first trimester remains the same as before gestation. The lack of pre-gestional evaluation, which would represent the normal gait pattern of the sample, limits the discussion and conclusion of the study [2]. We found only one study comparing the biomechanical adaptations of gait in the first trimester of pregnancy with the period before gestation [5]. However, as we mentioned in our review [4], the size of a sample determines the reliability and validity of the results, thus it is not possible to draw more general conclusions when the results are based on only few cases. Since the study was based on two subjects, our purpose was to verify the thesis on the schema of gait in early pregnancy on a larger group (15 subjects). Therefore, the aim of our research was to examine if the changes in the motor apparatus following the foetus development are recognizable in the first trimester of pregnancy, namely, if the parameters of early pregnant women are different than the ones observed before gestation. Branco et al. [20] emphasized a need to examine closely the kinematics of woman while walking, considering the beginning of pregnancy, in order to confirm the influence of morphological changes on the angular motion of the lower limb segments during the course of pregnancy.

The inclusion of anthropometric data may contribute to the analysis of its influence on biomechanical parameters [20]. From among the analysed anthropometric parameters, the significant changes in the analysed period were registered in circumferential features of soft tissues in the abdominal area and values of external conjugate of pelvis indicating at initial phase of external anatomic structures adaptation to the changes occurring in the reproductive system. Although Branchtein et al. [21] stress that influence of uterine volume on waist measurement appears to become important when fundal height reaches 27 cm, which corresponds to the median fundal height in 28^th^ week of gestation [22], in the group of the examined women the waist circumference and indexes based on it significantly changed. A study carried out by Sunaga et al. [7] recognized that physical changes during pregnancy are attributable to increased abdominal values thus even though the abdominal volumes of pregnant women in the first trimester appear small, the motion pattern differs from nulliparous women.

Authors emphasize that mass gain in pregnancy has the importance in the adaptations in gait mechanics associated with stability [23]. In our study we did not register mass gain between the two experimental sessions. Consequently, the analysis of gait pattern did not reveal any significant changes in terms of spatio-temporal parameters. The manner in which women were performing steps was not altered during early pregnancy. Therefore we confirmed the observation by Hagan and Wong [5], who noticed that stride length and gait speed were similar. Also joint kinematics remained similar throughout all phases of gait in both studies. No apparent changes occurred between pre-pregnancy and the period of 12^th^ to 16^th^ weeks, suggesting that peak relaxin level does not coincide with the observed gait changes [5]. Some authors noticed a reduced velocity [5, 10, 24], stride length [5, 8, 24, 25] or cadence [10, 24, 25] in the course of pregnancy while others did not observe significant changes of the parameters [8, 23, 26].

A combination of hormonal and biomechanical factors is responsible for providing a pelvic girdle stability. The pubic symphysis and sacroiliac joints widen in preparation for delivery. The pubis symphysis begins to widen between 10^th^ -12^th^ weeks of pregnancy while the joint width is normally 3–5 mm, it can become 5–8 mm during pregnancy [27]. Although we did not observe in our research any significant changes in the width dimension of the pelvis, we noted that the women modified the way of placing their feet on the ground. Thus our observation revealed a significantly larger ankle separation width. A widened stance is generally preferred during pregnancy [8] to increase the base of support [1]. It seems that the qualitative basis of these changes is our ability to naturally adapt the control system to the changing conditions that are necessary due to the need for efficient locomotion [28]. Authors emphasized that adaptations such as increased step width are recognised adopted strategy to provide safety and stability [29]. An increase in stance width allows to compensate increased mediolateral ground reaction force [30]. Enlargement of the area of support during walking may be also attributed to the increase of the body weight and pelvic size in advanced pregnancy [1]. Nevertheless, step width is also affected by trunk segment kinematics which can be altered as pregnancy progresses, as well as by mechanical obstruction when the girth of the thigh is increased. Gilleard believes that in this case the changes seen in step width may be of mechanical rather than functional origins [29].

Foti et al. [1] think that it is important to consider not only base of support dimension but also its relation to the pelvic width. In the research of Foti et al. the size of the base of support (mean ankle separation width during double support phase) and pelvic width significantly increased in terms of absolute values but the ratio of ankle width to the pelvic width (0.68 in pregnancy; 0.70 postpartum) remained constant. Following Foti et al. we also assessed standardized ankle width, however, in our study we did not observe a significant difference in terms of relative values. The above listed observations may confirm a safe strategy of the movements performed by women throughout their pregnancy period identified by some authors [1, 9, 10].

While the feet may adequately support and distribute body weight prior to carrying pregnancies, alterations during pregnancy can disrupt these supportive structures. Pregnancy-related increased ligamentous laxity, weight gain and shift in the centre of pressure can result in reduction in height of the longitudinal and transverse arches [31]. Ojukwu et al. reported high prevalence of low-arched feet among pregnant women [32]. On the contrary, Jelen et al. observed that it is not clear if pregnancy causes increasing or reducing of the plantar arch height, however, the study included only 4 women [33]. In the present study, no statistically important change in medial longitudinal arch shape was found.

According to Ribeiro et al., one of the pregnancy-related gait adaptations to improve stability is shift of the foot loading from the rearfoot to the midfoot and forefoot [9]. Also Karadag-Saygi et al. reported increased forefoot loading during pregnant women gait [34]. However, Golberg et al. research results showed otherwise i.e. increased plantar pressure in the rearfoot and reduced in the forefoot [35]. In our study forefoot and rearfoot loading ratio analysis showed no statistically important changes between women before pregnancy and in the 1^st^ trimester of gestation.

Another pregnancy-related adjustment which is believed to improve the postural stability control is gradual widening of the base of support [13]. According to Nyska et al., Goldberg et al. and Ribeiro et al. a greater base of support results in increased contact area of the foot with the ground [8, 35, 36]. In the present study, although wider base of foot support was observed, no increase of the foot contact area was found in the 1^st^ trimester of pregnancy comparing to pre-pregnancy period. The present study results showed that plantar pressure during gait is not altered throughout the first trimester of gestation. Possibly, the first trimester of pregnancy is too early to observe any foot loading changes, which can be explained by Alvarez et al. opinion that pregnancy-related foot loading alterations can be mainly attributed to increased foot volume (retention of liquids, increase of soft tissue) [37] observed in later stages of pregnancy.

A correction of the foot alignment may be used for the correct alignment of the pelvis. Authors emphasized that an increase of anterior tilt of the pelvis may lead to an increase of loads on the facet joints, which could result in lumbar back pain (e.g. [38]). Thus, for better understanding of the influence of foot positions on the kinematic chain, we also checked the behaviour of pelvic segment, since it is assumed that its changes can possibly lead to alterations of the spinal posture, in particular of the lumbar lordosis [39]. We measured the amplitude of the pelvis movements and it turned out that only transverse plane pelvis rotations were significantly reduced during early pregnancy. At the same time there were no significant alterations in the pelvic range of motion in sagittal and coronal plane. Then, observation of the instantaneous values of pelvic angles throughout gait cycle revealed significant differences in the pelvic obliquity and rotation. Our results showed that pregnancy limits pelvic rotations at the beginning and at the end of stance phase as well as throughout half of swing phase. Our research results showed that gestation also affects angular changes in the coronal plane when the supportive limb is load bearing (mid stance) for a short moment in last phase of single support. However, Hagan and Wong [5] reported lack of significant changes in the position of the pelvis throughout gait cycle in their two subjects. However, other authors observed more anteriorly tilted pelvis in the course of pregnancy [8, 25]. Saunders et al. [40] suggest that pelvis rotation and obliquity are the determinants of gait which reduces the movements of the centre of mass, thereby saving energy. Thus, our study results seem to indicate that changes in gait aiming to reduce energy expenditure are already taking place at an early stage of pregnancy.

Since our study is of longitudinal character, in the course of pregnancy we expect more clearly demonstrated changes in the movements of pelvis and other compensatory mechanisms. Due to that we hope to identify a strategy of the gravid body progression in space.

## Supporting information

S1 File Anthropometry data. BH—body height, BW—body weight, MUAC—mid-upper arm circumference, WC—waist circumference, HC—hip circumference, IC-IC–intercristal diameter, IS-IS–interspinous diameter, EC–external conjugate diameter, BMI—Body mass index, WHR–waist to hip ratio, WHtR–waist to height ratio, TER—trunk to extremity ratio, %FAT—percentage of body fat.
(XLSX)

S2 File Gait data. Cad-Cadence, DS-Double Support, SS -Single Support, FO—Foot Off, OFC -Opposite Foot Contact, OFO -Opposite Foot Off, SL -Stride Length, ST -Stride Time, v—velocity, Pelvic ranges of motion (ROM), IAD—inter-ankle distance; IMD—inter 5th metatarsal distance; N IAD—normalized inter-ankle distance.
(XLSX)

S3 File Foot data. DAI—dynamic longitudinal arch index according to Cavanagh (R / L—right / left foot), FRI—forefoot [%] and rearfoot [%] loading ratio (R / L—right / left foot), MLI—ratio of medial [%] and lateral [%] foot loading (R / L—right / left foot), CA—an average contact area [cm^2^] (R / L—right / left foot), MP—an average maximal pressure [gr/cm2] (right / left foot).
(XLSX)
